# Malignant Hyperthermia in Sweden: Clinical Presentations and Genetic Findings

**DOI:** 10.1111/aas.70255

**Published:** 2026-05-13

**Authors:** Anna Hellblom, Maria Soller, Carolina Samuelsson

**Affiliations:** ^1^ Department of Intensive and Perioperative Care Skåne University Hospital Lund Lund Sweden; ^2^ Department of Laboratory Medicine, Clinical Genetics Lund University Lund Sweden; ^3^ Department of Molecular Medicine and Surgery Karolinska Institutet Stockholm Sweden; ^4^ Department of Clinical Sciences, Anaesthesiology and Intensive Care Lund University Lund Sweden; ^5^ Region Halland (the Regional Council of Halland County) Halland Sweden

## Abstract

**Introduction:**

Malignant hyperthermia (MH) is a pharmacogenetic, hypermetabolic and potentially lethal reaction to potent volatile anaesthetics and the muscle relaxant succinylcholine. To improve the understanding of MH, the aim of this retrospective study was to describe the Swedish cohort with respect to clinical manifestations, demographics and genetic findings.

**Method:**

The Swedish MH Registry covers a total of 2852 individuals belonging to 544 different families investigated for MH since 1980. For this study, the index case in each family was included for further description.

**Results:**

In total, MH was confirmed in 1555 individuals. Among the 288 index cases with a history of an MH reaction and confirmed MH, 58% were male and 57% were < 18 years of age. MH was confirmed in 41% of index cases where masseter muscle spasm was the only clinical sign of MH. The overall case fatality following an MH reaction in Sweden was 5.2%, but no fatal MH reaction has been reported since 2001. Out of 163 genetically investigated MH families, 61 had a diagnostic variant and 23 had a variant of unknown significance.

**Conclusions:**

We found that MH reactions predominantly occur in young individuals and that case fatality has declined over recent decades, with no deaths reported in the past 20 years. Less than 40% of the genetically investigated MH families, corresponding to around 20% of all Swedish MH families, currently have an identified genetic diagnostic variant.

**Editorial Comment:**

This analysis of the national Swedish Malignant Hyperthermia patient registry presents case factors and outcomes from cases among recognised families from the last 45 years. Presenting symptoms and then case confirmation details are presented, along with results from cases and families where genetic variants has been assessed.

## 1 Introduction

**FIGURE 1:**
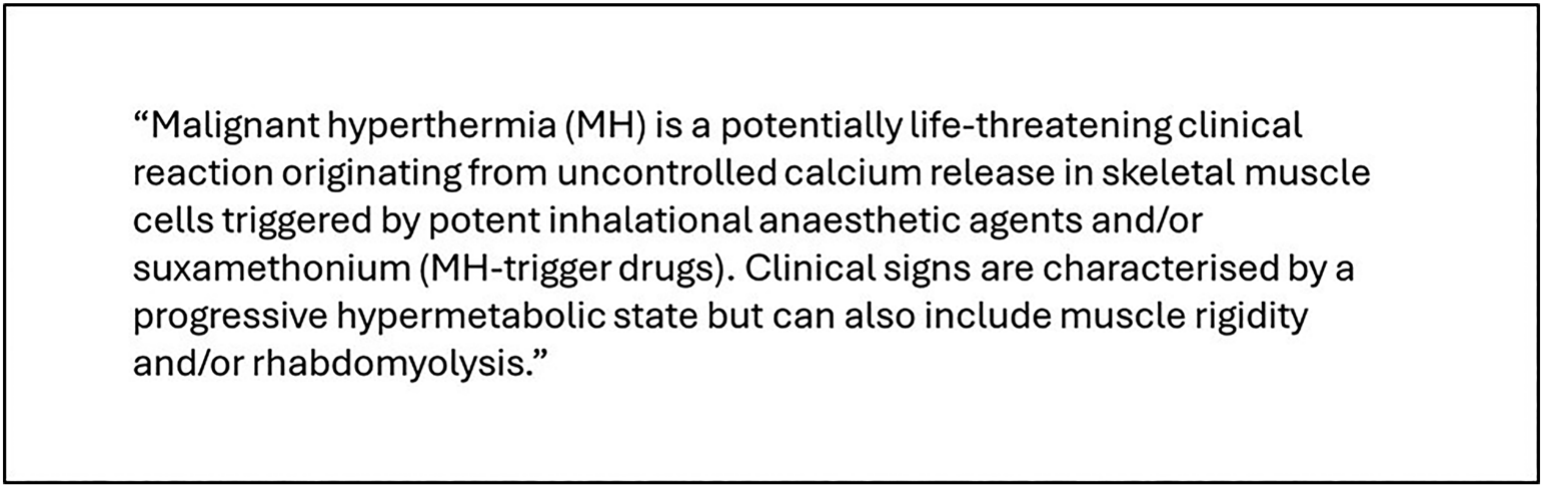
*Source:* Rüffert et al. [2].

In 1962, the first case report of a life‐threatening reaction to general anaesthesia was published [1]. In Australia, a 21‐year‐old male with a family history of 10 deaths during general anaesthesia developed tachycardia, low blood pressure, cyanosis and hyperthermia during halothane anaesthesia. This adverse event is now known as a clinical reaction of malignant hyperthermia (MH)—a pharmacogenetic, hypermetabolic and potentially lethal reaction to potent volatile anaesthetics and the muscle relaxant succinylcholine (Figure 1).

In the 1970s, the mortality associated with an MH reaction was high, and most patients did not survive. The introduction of dantrolene in the 1970s, combined with improved anaesthetic monitoring and systematic family investigations, has markedly reduced the mortality and is now commonly reported to be approximately 5%–10% [3, 4, 5, 6].

Investigation for MH can include a muscle contracture test, a genetic investigation or both. A contracture test is the gold standard for diagnosing or ruling out MH susceptibility (MHS). The in vitro contracture test (IVCT), performed according to the European Malignant Hyperthermia Group (EMHG) protocol [2], is the most common test used worldwide and has a sensitivity of 99% and specificity of 94% [7].

An alteration in one of three genes is associated with MH, all of which are involved in the excitation‐contraction coupling of skeletal muscle cells. The variants in these genes that are classified as pathogenic or likely pathogenic for MH are used to assess the MH genotype (MHG). They are sometimes referred to as ‘diagnostic variants’. The *RYR1* gene, encoding the skeletal muscle sarcoplasmic reticulum calcium release channel, is accountable for most diagnostic MH variants. Two diagnostic variants are identified in *CACNA1S*, the gene encoding the dihydropyridine receptor [8]. The autosomal recessive STAC3 myopathy has also been associated with MH [9].

Despite increasing knowledge of the genetic background of MH, a significant proportion of MH families still lack a genetic explanation for their condition [10, 11]. In addition, a European multicentre study showed that in about 8% of families, there is discordance in at least one individual between the genetic result and the IVCT [11]. If, in families with an MH reaction, the diagnostic variant is absent in a family member, an IVCT must be performed in order to rule out MH.

Traditionally, the indication for referral for MH diagnostics is a personal or a family history of a suspected MH reaction during general anaesthesia. Over time, other indications where patients could be at increased risk of MH have evolved. These indications mainly include *RYR1*‐associated congenital myopathies, the most common being central core disease or the finding of a *RYR1* variant of unknown significance (VUS). The availability of next‐generation sequencing, that is, the possibility for screening of multiple genes in one analysis, has, in the last decade, led to increased referrals for patients without a history of a suspected MH reaction [12]. The updated EMHG diagnostic guidelines from 2025 advise differentiated diagnostic pathways depending on whether there is a history of a suspected MH reaction or not [2].

To improve the understanding of MH, the aim of this retrospective study was to describe the Swedish cohort with respect to clinical manifestations, demographics and genetic findings. Among the genetic findings not already classified as diagnostic for MH, we aimed to identify variants that may be of interest to investigate further.

## 2 Materials and Methods

The Swedish MH Registry covers all investigated individuals referred from Swedish health care providers since 1980. By the end of 2024, a total of 2852 individuals belonging to 544 different families were registered. For this study, the index case in each family was included for further description.

### 2.1 Clinical Evaluation of Index Cases

Information from the registry and available medical records of the index cases were reviewed to retrieve referral details and demographic data. A quality check of the registry entries was made to complete missing information and ensure correctness. For those with available clinical information from a suspected MH reaction, the Clinical Grading Score by Larach (Larach score) [13] was calculated. This grading score is based on assessment of indicators of the different processes of an MH reaction (e.g., muscle rigidity, signs of muscle breakdown, respiratory acidosis, rise in temperature and cardiac involvement), which, when summarised, gives scores ranging from 0 to 88. The raw score is used to assign each index case an MH rank of 1 (*almost never*) to 6 (*almost certain*), reflecting the likelihood of MH. For this study, index cases with a fatal reaction were assigned an MH rank of 6^†^.

### 2.2 Diagnostic Tests

The MH Unit in Lund has consistently adhered to the most recently published EMHG diagnostic protocol [14, 15, 16]. Before the first EMHG protocol, testing was conducted according to the procedure described by Ørding et al. [17]. For the purpose of this study, the term ‘confirmed MH’ refers either to MHS diagnosed by IVCT or an MHG. In analogy, an ‘MH family’ is defined as a family with at least one individual in whom MH has been confirmed.

#### 2.2.1 IVCT

For IVCT, muscle specimens were exposed to increasing concentrations of halothane and caffeine separately while measuring the muscle contraction. A normal response to both substances classifies the patient as MH negative (MHN), and a pathological response to both or either one of the substances classifies the patient as MHS.

#### 2.2.2 Genetic Diagnostic Techniques and Classification Criteria

Genetic screening was performed on the index case when possible; otherwise, it was performed on an MHS family member. Any detected variant was assigned at the family level and designated as the familial variant.

The genetic techniques have advanced over time—from Sanger sequencing of *RYR1* mutation ‘hot spots’ to exome sequencing of *RYR1* and, more recently, to whole‐genome sequencing enabling analysis of multiple genes of interest. Classification of *RYR1* variants follows the EMHG scoring matrix [18], and the variants classified as pathogenic or likely pathogenic are accordingly defined as ‘diagnostic variants’. The remainder of variants in *RYR1*, not classified as normal variants (benign), are listed as VUS. The classification may differ from that of the American College of Medical Genetics and Genomics (ACMG) and the ClinGen Variant Curation Expert Panel (VCEP), as described in the EMHG guidelines [2].

### 2.3 Statistics

Descriptive demographic and diagnostic statistics for index cases are presented either as a proportion or as a median with interquartile range (IQR). A one‐tailed Mann–Whitney *U* test was used to calculate whether the MH rank was higher in the group of index cases with confirmed MH compared to those who were diagnosed with MHN. A chi‐square test was used to compare the diagnostic outcome between sexes. A *p* < 0.05 was considered statistically significant.

### 2.4 Ethical Approval

The study was approved by the Swedish Ethical Review Authority (2019‐03960).

## 3 Results

**FIGURE 2:**
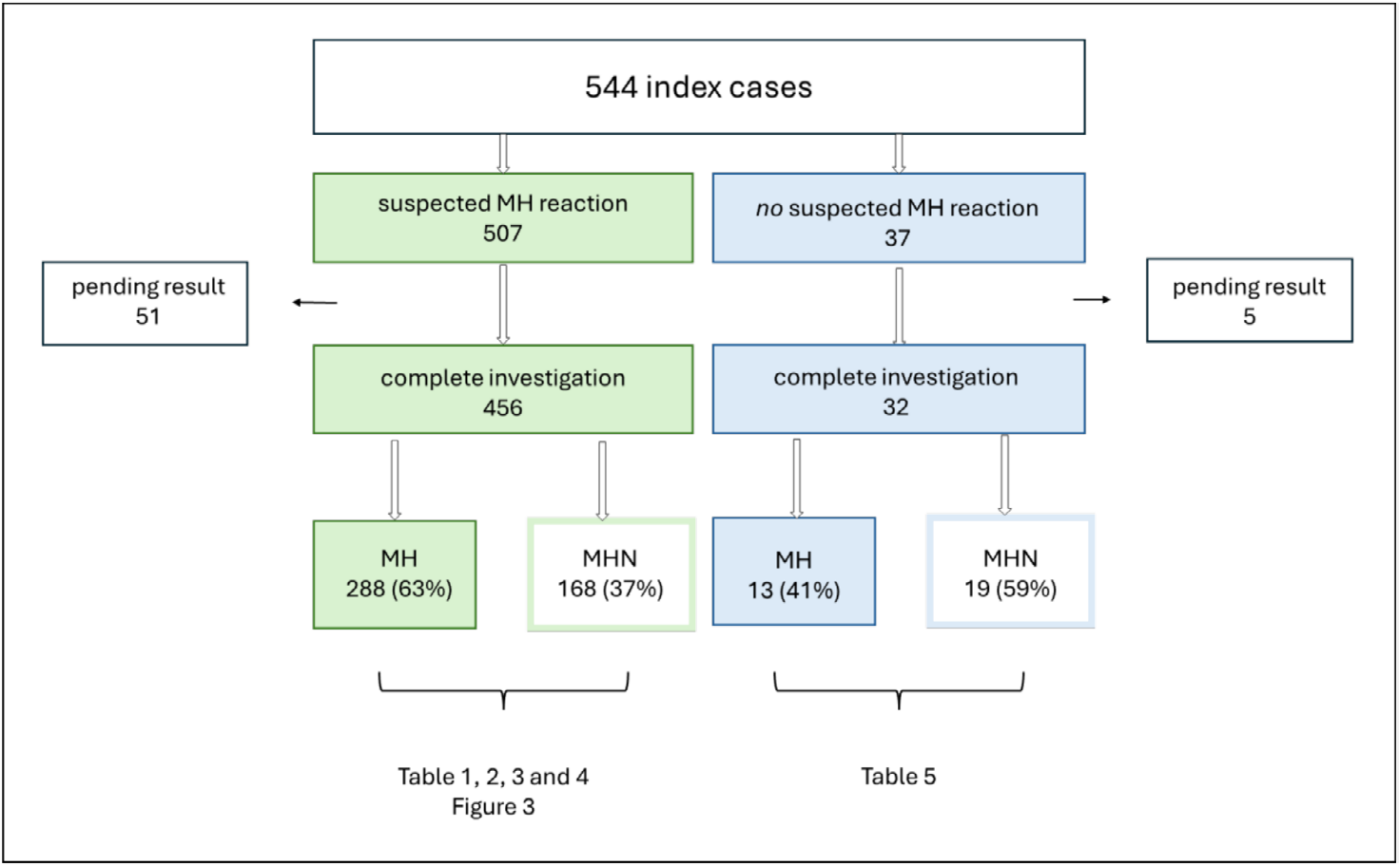
An outline of the 544 index cases, their referral indication and MH diagnostic results. Green: Index cases referred following a suspected MH reaction. Blue: Index cases referred for other reasons than a suspected MH reaction. MH, confirmed MH; MHN, MH negative.

Between 1980 and 2024, a total of 2852 individuals were investigated for MH, of whom 1555 received a confirmed MH diagnosis. Out of the 544 index cases, 507 were referred due to a suspected MH reaction and 37 for other reasons where an increased risk of MH could be suspected (Figure 2). Among the 507 index cases with a suspected MH reaction, 34 cases occurred outside of Sweden.

### 3.1 Index Cases With a Suspected MH Reaction

**FIGURE 3:**
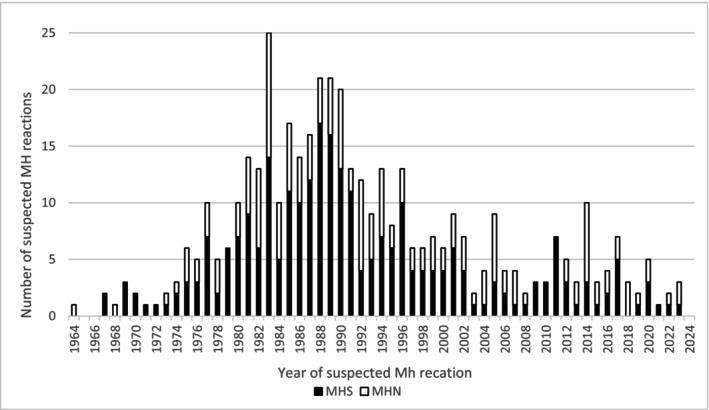
Distribution over the years for investigated index cases with a suspected MH reaction that occurred in Sweden and their MH status.

As presented in Figure 2, MH was confirmed in 288 of 456 (63%) index cases with a suspected MH reaction and a completed MH investigation. For 270 of these index cases, the reaction occurred in Sweden. Figure 3 illustrates the distribution of the suspected MH reactions that occurred in Sweden over a 60‐year time period, with the earliest case from 1964. Peaking in the late 1980s, the annual number of referrals has decreased and, over the past decades, has remained at 10 or fewer per year.

Details on the index cases with a suspected MH reaction—including demographics, Larach score and MH rank—are outlined in Table 1. Of the index cases with confirmed MH, 162 of 280 (58%) were male and 154 of 271 (57%) were < 18 years of age. Also, MH was confirmed in 162 of 224 (72%) referred males, a significantly higher proportion compared to 118 of 224 (53%) referred females (*p* < 0.001). The median Larach score in those with confirmed MH was 30 (IQR: 15–43) compared to 15 (IQR: 15–18) in the MHN group. These scores correspond to MH Rank 4 in index cases with confirmed MH compared with Rank 3 in the MHN group (*p* < 0.001). Data on sex, age and reaction details were missing for 8, 22 and 34 index cases, respectively. The clinical signs of index cases with confirmed MH are shown in Table 2. MH was confirmed in 48 of 116 (41%) index cases where masseter muscle spasm was the *only* clinical sign of MH. There was a fatal outcome in 14 of the 270 MH reactions that occurred in Sweden during the studied period. This corresponds to an overall case fatality of 5.2%. However, no fatal MH reaction has been reported in Sweden since 2001. Of the 14 deaths, 11 occurred in patients under the age of 30 years.

**TABLE 1:** Demographics and clinical data of the index cases with a suspected MH reaction, presented for MH and MHN separately.

	MH (*N* = 288)	MHN (*N* = 168)
Sex		
Female	118/280 (42%)	106/168 (63%)
Male	162/280 (58%)	62/168 (37%)
Age group		
0–12	130/271 (48%)	30/163 (18%)
13–17	24/271 (9%)	17/163 (10%)
18–30	60/271 (22%)	54/163 (33%)
> 30	57/271 (21%)	62/163 (38%)
Larach Clinical Grading Score		
Median (IQR)	30 (15–43)	15 (15–18)
MH rank		
1—*Almost never*	1/264 (0.5%)	10/158 (6%)
2—*Unlikely*	1/264 (0.5%)	8/158 (5%)
3—*Somewhat less than likely*	94/264 (36%)	103/158 (65%)
4—*Somewhat greater than likely*	66/264 (25%)	25/158 (16%)
5—*Very likely*	58/264 (22%)	12/158 (8%)
6—*Almost certain*	30/264 (11%)	0/158
6^†^—*Death*	14/264 (5%)	0/158
Median MH rank*	4	3

Abbreviations: MH, confirmed MH; MHN, MH negative.

* *p* < 0.001, Mann–Whitney *U* test.

**TABLE 2:** Clinical signs of MH in 259 index cases with confirmed MH.

Signs of an MH reaction	
Masseter spasm	164/259 (63%)
Generalised muscle rigidity	82/259 (32%)
Increased CO_2_/tachypnea	106/259 (41%)
Sinus tachycardia	132/259 (51%)
Muscle breakdown	110/259 (42%)
Rise in temperature	95/259 (37%)
BE< −8 or pH < 7.25	45/259 (17%)
VT/VF	16/259 (6%)

Abbreviations: VF, ventricular fibrillation; VT, ventricular tachycardia.

Genetic screening was performed in 163 of the 288 MH families. In 84 (52%) of these families, a *RYR1* variant was identified; 61 of these were diagnostic for MH. Thus, overall, 61 of 163 (37%) genetically investigated families, and 61 of 288 (21%) Swedish MH families in total had a diagnostic variant. The *RYR1* variants in the remaining 23 of 163 (14%) families were classified as VUS. There was no carrier of a CACNA1S or STAC3 variant in the entire cohort. More detailed descriptions of the genetic findings in the MH families are shown in Tables 3 and 4.

**TABLE 3:** Pathogenic and likely pathogenic *RYR1* variants for 61 MH families with an MH reaction.

Exon	Nucleotide change	Amino acid change	Classification by EMHG	No. of families
1	c.38T>G	p.Leu‐13‐Arg	P	1
6	c.487C>T	p.Arg‐163‐Cys	P	1
9	c.742G>C	p.Gly‐248‐Arg^a^	P	4
11	c.1024C>A	p.Glu‐342‐Lys^b^	P	1
15	c.1589G>A	p.Arg‐530‐His	LP	1
15	c.1654C>T	p.Arg‐552‐Trp	P	2
17	c.1840C>T	p.Arg‐614‐Cys	P	7
17	c.1841G>T	p.Arg‐614‐Leu	P	1
39	c.6487C>T	p.Arg‐2163‐Cys	P	3
39	c.6502G>A	p.Val‐2168‐Met	P	2
40	c.6617C>G	p.Thr‐2206‐Arg	P	1
40	c.6617C>T	p.Thr‐2206‐Met	P	2
42	c.6863T>C	p.Leu‐2288‐Ser	P	1
43	c.7007G>A	p.Arg‐2336‐His	P	3
44	c.7042_7044del	p.Glu‐2348‐del	P	1
44	c.7063C>T	p.Arg‐2355‐Trp	P	7
45	c.7300G>A	p.Gly‐2434‐Arg	P	8
45	c.7304G>A	p.Arg‐2435‐His	P	7
46	c.7360C>T^c^	p.Arg‐2454‐Cys^c^	P	1
46	c.7361G>A	p.Arg‐2454‐His	P	1
46	c.7373G>A	p.Arg‐2458‐His	P	1
47	c.7523G>A	p.Arg‐2508‐His	P	2
50	c.8026C>T	p.Arg‐2676‐Trp	LP	1
101	c.14,545G>A	p.Val‐4849‐Ile	P	2

Abbreviations: LP, likely pathogenic variant; P, pathogenic variant.

^a^ In one family, p. Arg‐1583‐Cys (VUS) was also detected.

^b^ In one family, p. Glu‐572‐Ala (VUS) was also detected.

^c^ Homozygous.

**TABLE 4:** *RYR1* variants of unknown significance (VUS) in MH families with a clinical MH reaction.

Exon	Nucleotide change	Amino acid change	Classification by EMHG	No of families
3	c.178G>A	p.Asp‐60‐Asn	VUS	1
11	c.982C>T	p.Arg‐328‐Trp	VUS	1
12	c.1144C>A	p.His‐382‐Asn	VUS	1
15	c.1597C>A	p.Arg‐533‐Ser^a^	VUS	1
24	c.3172G>A	p.Glu‐1058‐Lys	VUS	1
29	c.4178A>G	p.Lys‐1393‐Arg	VUS	1
34	c.5036G>A	p.Arg‐1679‐His^b^	VUS	1
39	c.6544A>T	p.Ile‐2182‐Phe	VUS	1
40	c.6599C>T	p.Ala‐2200‐Val	VUS	1
44	c.7099G>A	p.Ala‐2367‐Thr	VUS	1
46	c.7373G>T	p.Arg‐2458‐Leu^a^	VUS	1
47	c.7522C>G	p.Arg‐2508‐Gly^a^	VUS	1
49	c.7854G>A	p.Met‐2618‐Ile	VUS	1
60	c.9101A>C	p.Lys‐3034‐Thr	VUS	2
66	c.10010G>A	p.Arg‐3337‐Gln	VUS	1
69	c.10359C>T	p.Arg‐3453=	VUS	1
71	c.10616G>A	p.Arg‐3539‐His	VUS	2
87	c.11958C>G	p.Asp‐3986‐Glu	VUS	1
89	c.12122G>A	p.Arg‐4041‐Gln	VUS	1
101	c.14539G>C	p.Val‐4847‐Leu	VUS	1
46 + 102	c.7433C>A + c.14782A>G	p.Thr‐2478‐Asn + p.Ile‐4928‐Val	VUS	1

Abbreviation: VUS, variant of unknown significance.

^a^ Amino acid change in the same position as a variant classified as pathogenic.

^b^ This amino acid change was also found in an MHN patient.

In the 23 families, there were 21 unique VUS. Eleven of these were located in the same exons as diagnostic variants. Two *RYR1* VUS, p.Arg‐3539‐His and p.Lys‐3034‐Thr, were each identified in two unrelated MH families. Another three VUS had an amino acid change in the same position as variants already classified as pathogenic or likely pathogenic.

Out of the 48 index cases with masseter muscle spasm and confirmed MH, 20 had a genetic investigation. Of these, two had a diagnostic *RYR1* variant, and another five had an *RYR1* VUS.

In 10 of 288 (3.5%) MH families and a total of 17 individuals, there was discordance between the genetic result and the following IVCT. In two families, there was a discordance in more than one family member. These 17 individuals lacked the familial, diagnostic *RYR1* variant but were diagnosed with MHS by IVCT.

### 3.2 Index Cases Without a Suspected MH Reaction

Out of the 37 index cases referred for MH investigation without a suspected clinical reaction, MH was confirmed in 13 (41%) and ruled out in 19 (59%). For five index cases, the MH status is still pending (Figure 2). The most common referral indication in this group was central core disease. All referral indications and MH status for those without an anaesthetic reaction are shown in Table 5.

**TABLE 5:** Referral indications for index cases without suspected MH reaction.

Diagnosis	MH (*N* = 13)	MHN (*N* = 19)
Central core disease	5	3
Multi‐minicore	1	0
Myopathy/hyperCK	3	2
Rhabdomyolysis	1	2
Heat Stroke	0	2
Hypokalaemia periodic paralysis	0	1
*RYR1* VUS	0	4
Incidental diagnostic *RYR1*	2	0
CACNA1S VUS	0	1
Other	1	4

Abbreviations: MH, confirmed MH; MHN, MH negative.

## 4 Discussion

This study is, to date, the most comprehensive description of the Swedish MH cohort, outlining clinical features of index cases, genetics of the Swedish MH families and potential genetic variants to investigate further. We found that over the past 60 years, there were 270 confirmed MH reactions in Sweden, 14 of them with a fatal outcome. Consistent with previous reports [4, 19, 20, 21], we found MH reactions predominantly in younger individuals and with a male preponderance. The higher proportion of confirmed MH among referred males compared with females (72% vs. 53%) was somewhat unexpected. As referral criteria were identical for both sexes, this difference is unlikely to be due to referral bias and may reflect the male preponderance. Referrals for suspected MH reactions have decreased over time, with a current rate of 10 or fewer referrals per year. This may be due to the decreasing use of volatile anaesthetics and succinylcholine.

In Sweden, the overall case fatality of an MH reaction was 5%, with no lethal reactions reported since 2001. This supports previously published data that lethal MH reactions are decreasing [5]. It is still important to understand that an MH reaction can occur in *any* patient, including the young and otherwise healthy. Knowledge of how to manage an MH reaction must therefore be part of any institution delivering general anaesthesia. Nonetheless, we have recently published data indicating that there is still room for improvement in MH‐related preparedness and patient safety among Swedish surgical care providers [22].

Interestingly, as many as 41% of those referred for MH investigation with the only clinical sign being masseter muscle spasm were diagnosed with MH. This is a higher proportion than the 28% previously reported by Ellis et al. [23]. Contraction of the masseter muscle is a normal response to succinylcholine, and common definitions are ‘a marked and prolonged muscle contraction that may interfere with intubation’ or ‘jaws of steel’. In clinical practice, however, the identification of masseter muscle spasm relies on a subjective perception of the anaesthetist and may lead to a change in anaesthetic management without triggering agents that change the clinical course. Other conditions besides MH, such as myotonic dystrophy and myotonia congenita, are also associated with succinylcholine‐induced masseter muscle spasm. Our finding underscores that, unless these myopathies are apparent, referral for MH investigation is indicated.

The MH rank, based on the Larach score, was significantly higher in the index cases with confirmed MH compared to those who were MHN. The Larach score may be underestimated when relevant clinical or biochemical parameters are unavailable. Also, early modification of anaesthetic management—such as withdrawal of triggering agents in response to masseter muscle spasm—may alter the clinical course and thereby influence the scoring. With this in mind, a score difference of 15 may not be of clinical relevance, and it is important to point out that the Larach clinical grading scale was originally developed for research purposes to create a clinical case definition and not as a clinical decision tool to determine whether to investigate for MH or not.

We found that only about 20% of all the Swedish MH families have a diagnostic variant and could therefore benefit from the genetic and less invasive investigation. Among the genetically investigated MH families, 36% had a diagnostic variant and another 14% had a VUS. These findings align with previous studies, reporting ranges of 37%–76% [10, 24, 25]. However, since many MH families were never subjected to next‐generation sequencing, the actual proportion may be underestimated. The IVCT is an invasive test and for most patients implies travelling, a few days' absence from school or work and refraining from more extensive physical activity for up to 2 weeks. In addition, an IVCT is more expensive and less accessible than a genetic test. With increased coverage and a deeper understanding of genetics, more patients could benefit from a genetic test and be diagnosed without an invasive procedure.

Most pathogenic and likely pathogenic MH variants are missense mutations in *RYR1*, in line with earlier genetic findings reported for the Swedish MH cohort [26]. Missense mutations typically alter charge or polarity, potentially affecting calcium channel function. All VUS in the present cohort were missense mutations, and about half of them are located in the same exons as the diagnostic variants. This suggests that a substantial portion of the Swedish VUS pool shares structural and positional features with known MH variants. Functional studies and co‐segregation analyses could help reclassify some of these VUS as pathogenic or likely pathogenic. The *RYR1* variants p.Arg3539His and p.Lys3034Thr warrant further evaluation, as their identification in two unrelated MH families provides supportive evidence for a potential pathogenic role. The three variants that result in an amino‐acid substitution at the same position as variants already classified as pathogenic are also potential candidates for genetic curation. No pathogenic variants in CACNA1S or STAC3 were detected, consistent with their infrequent involvement in MH.

The EMHG diagnostic guidelines state that the absence of the familial, diagnostic variant is not enough to rule out MH in family members where the index case had an MH reaction, since large pedigree studies have shown discordance between genetic results and IVCT [2, 10, 11]. This discordance may be explained by a false‐positive IVCT result or alternatively reflect the presence of more than one genetic risk factor. Our finding of discordance in 17 cases emphasises the need for an IVCT after a negative genetic test for maximal anaesthetic safety.

In Sweden, the majority of index cases investigated for MH were referred because of a suspected MH reaction. However, of the 32 index cases investigated for reasons other than a suspected MH reaction, about 40% were MHS. A recent multicentre study, which included data from Sweden, showed a similar proportion of confirmed MH among patients referred without a history of suspected MH reaction [12]. Compared with the other MH centres, Lund had a smaller proportion of referrals without a suspected MH reaction. The same study also showed that with increasing availability to next‐generation sequencing techniques and the finding of *RYR1* VUS in the workup of neuromuscular disease, there has been an increase in referrals for MH investigation of patients without an anaesthetic adverse event.

### 4.1 Strengths and Limitations

Our 45‐year‐old registry, covering all patients investigated for MH in Sweden, provides a unique opportunity to study MH. Nevertheless, there are limitations related to the retrospective study design, especially as it encompasses a long period of time. Even though we made efforts to obtain data integrity, there was still missing information from some index cases and their MH reaction. In addition, the diagnostic results for some recently referred patients are still pending due to limited IVCT capacity. Also, over the years, the decision to investigate index cases without a suspected MH reaction may have differed.

## 5 Conclusion and Unanswered Questions

MH reactions are potentially life‐threatening events that predominantly occur in young individuals. Importantly, case fatality has declined over recent decades, and no deaths have been reported in the past 20 years.

Despite advances in genetic diagnostics, only 37% of the tested MH families and about 20% of all Swedish MH families currently have an identified diagnostic variant. Expanding the genetic understanding of MH within the Swedish cohort is therefore essential, particularly regarding the *RYR1* variants of unknown significance. Improved classification of these variants could enable a larger proportion of patients to receive a genetic diagnosis and thereby avoid the need for IVCT.

## Author Contributions

**Anna Hellblom:** conceptualisation, data curation, formal analysis, investigation, methodology, visualisation, writing – original draft, writing – review and editing. **Maria Soller:** conceptualisation, methodology, supervision, writing – review and editing. **Carolina Samuelsson:** conceptualisation, funding acquisition, methodology, supervision, writing – review and editing.

## Funding

This work was supported by the Anna and Edwin Bergers Foundation.

## Conflicts of Interest

The authors declare no conflicts of interest.

## Data Availability

The data that support the findings of this study are available from the corresponding author upon reasonable request.
